# Whole-Exome Sequencing Identifies One De Novo Variant in the *FGD6* Gene in a Thai Family with Autism Spectrum Disorder

**DOI:** 10.1155/2018/8231547

**Published:** 2018-05-17

**Authors:** Chuphong Thongnak, Areerat Hnoonual, Duangkamol Tangviriyapaiboon, Suchaya Silvilairat, Apichaya Puangpetch, Ekawat Pasomsub, Wasun Chantratita, Pornprot Limprasert, Chonlaphat Sukasem

**Affiliations:** ^1^Division of Pharmacogenomics and Personalized Medicine, Department of Pathology, Faculty of Medicine Ramathibodi Hospital, Mahidol University, Bangkok, Thailand; ^2^Laboratory for Pharmacogenomics, Somdech Phra Debaratana Medical Center (SDMC), Ramathibodi Hospital, Bangkok, Thailand; ^3^Department of Clinical Pathology, Faculty of Medicine Vajira Hospital, Navamindradhiraj University, Bangkok, Thailand; ^4^Graduate Program in Biomedical Sciences, Faculty of Medicine, Prince of Songkla University, Songkhla, Thailand; ^5^Rajanagarindra Institute of Child Development, Chiang Mai, Thailand; ^6^Division of Pediatric Cardiology, Department of Pediatrics, Faculty of Medicine, Chiang Mai University, Chiang Mai, Thailand; ^7^Excellence Center for Genomic Medicine, Faculty of Medicine Ramathibodi Hospital, Mahidol University, Bangkok, Thailand; ^8^Virology and Molecular Microbiology Unit, Department of Pathology, Faculty of Medicine Ramathibodi Hospital, Mahidol University, Bangkok, Thailand; ^9^Human Genetics Unit, Department of Pathology, Faculty of Medicine, Prince of Songkla University, Hat Yai, Songkhla, Thailand; ^10^Faculty of Medicine, King Mongkut's Institute of Technology Ladkrabang, Bangkok, Thailand

## Abstract

Autism spectrum disorder (ASD) has a strong genetic basis, although the genetics of autism is complex and it is unclear. Genetic testing such as microarray or sequencing was widely used to identify autism markers, but they are unsuccessful in several cases. The objective of this study is to identify causative variants of autism in two Thai families by using whole-exome sequencing technique. Whole-exome sequencing was performed with autism-affected children from two unrelated families. Each sample was sequenced on SOLiD 5500xl Genetic Analyzer system followed by combined bioinformatics pipeline including annotation and filtering process to identify candidate variants. Candidate variants were validated, and the segregation study with other family members was performed using Sanger sequencing. This study identified a possible causative variant for ASD, c.2951G>A, in the *FGD6* gene. We demonstrated the potential for ASD genetic variants associated with ASD using whole-exome sequencing and a bioinformatics filtering procedure. These techniques could be useful in identifying possible causative ASD variants, especially in cases in which variants cannot be identified by other techniques.

## 1. Introduction

Autism spectrum disorder (ASD) is a neurodevelopmental disorder described by abnormalities in two domains: (1) social interaction and communication/language and (2) restricted and repetitive behavior. A study of the prevalence of autism in Thailand showed that it affects 9.9 children per 10,000 populations for children 1–5 years old [1]. According to the *Diagnostic and Statistical Manual of Mental Disorders, Fifth Edition (DSM-5)*, autism subtypes, autistic disorder, Asperger's disorder, pervasive developmental disorder not otherwise specified (PDD-NOS), and childhood disintegrative disorder (CDD), are merged to a single term “autism spectrum disorder.” Rett disorder that was previously assigned as an autism subtype under DSM-IV criteria was classified as a separate disorder [2, 3].

Autism disturbs information processing in the brain by changing connection and organization of nerve cells and their synapses, the cause of which is not well understood [4]. ASD has a strong genetic root, although the genetics of autism is unclear and complex whether autism can have explained more by major effects of rare mutations or by common genetic variants with rare multigene interactions [5, 6]. Interactions of several genes, epigenetic factors, and the environment increase the complexity of etiology and physiology in ASD [7]. A number of researchers have attempted to explore the genetic basis of autism to apply in diagnosis. Studies to find candidate genes that are associated with ASD have used cytogenetics, targeted sequencing, and genome-wide association; identifying a large amount of genes may be responsible [5, 8] such as *SHANK3* on chromosome 22p16.3 and *NRXN1* and *NLGN3/4* on chromosomes Xq13 and Xq22.33 [9]. Several candidate genes have been located but cannot explain genetic mechanisms of ASD.

High-throughput sequencing has less bias for common variants than other techniques; therefore, it is an effective tool to discover genetic variants at genome-wide level and capable to apply in personalized medicine [10]. Exome sequencing is one high-throughput sequencing application that has been widely used in medical research, especially to find disease candidate genes in the last decade [11, 12]. It can expedite diagnosis through the sequencing of all exon or coding regions of the genome simultaneously [13]. In the field of autism, exome sequencing was also used to find genetic causes or related variation [14–16]. These showed the potential and possibility of the exome sequencing applications in genetic study of autism.

This study aims at applying whole-exome sequencing in identifying possible autism causative variants in two Thai families. Three de novo variants were detected in one family by using analysis pipeline, which was executed by integration of familial data, exome sequencing, bioinformatics, and segregation analysis.

## 2. Materials and Methods

### 2.1. Subjects

**Figure 1:**
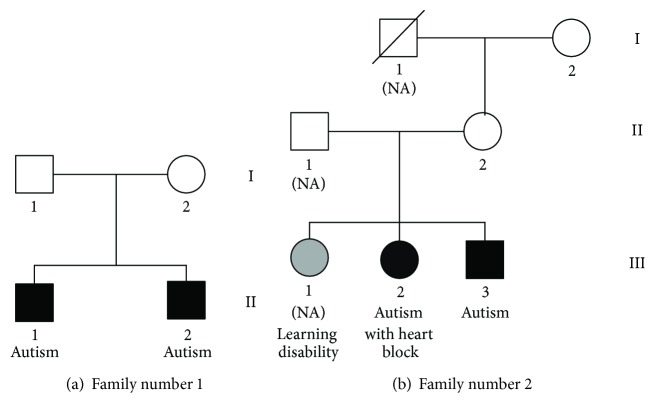
Pedigree of the family with autism indicated by the dark symbol. Gray symbol indicates learning disability phenotype. Females are indicated by circles and males by squares.

The subjects in this study were autism-affected children and normal individuals from two unrelated families. The family number 1 consisted of unaffected parents (1.I-1 and 1.I-2) and two children with autism (1.II-1 and 1.II-2). The proband (1.II-1) was featured in a previous cohort study [17]. The family number 2 had three generations, of whom four out of seven members were available for DNA study, an unaffected grandmother (2.I-2), an unaffected mother (2.II-2), and two children with autism (2.III-2 and 2.III-3). The proband of the family number 2 (2.III-2) had also been included in another study [18]. The clinical information of both families is shown in Supplementary 1. The pedigree for both families is shown in Figure 1. All samples had negative results when screened for common autism-related disorders in a previous microarray study. Genomic DNA was obtained from the Department of Pathology, Faculty of Medicine, Prince of Songkla University. All samples from patients were prepared for whole-exome sequencing while available samples from other family members were prepared for segregation analysis. Written informed consent was obtained from adult individuals themselves and for all children. All procedures were approved by the Institutional Review Board (MURA2012/02/SN1) of the Faculty of Medicine Ramathibodi Hospital, Mahidol University, and the Faculty of Medicine, Prince of Songkla University (EC48/364-006-3).

### 2.2. Exome Sequencing and Data Analysis

All samples passed quality verification by measurement of DNA concentration by Nanodrop ND1000 and measurement of DNA fragmentation by agarose gel electrophoresis. DNA samples of the samples 1.II-1, 1.II-2, 2.III-2, and 2.III-3 were prepared for whole-exome sequencing following standard SOLiD 5500xl (Applied Biosystems, California, USA) protocols. Each sample was diluted to three micrograms followed by fragmentation process using Covaris s220 (Covaris, USA) sonication machine. Agencourt beads were used to select for defined fragment size, which was measured using Agilent Bioanalyzer. TargetSeq Exome and Custom Enrichment System (Invitrogen, California, USA) was used to capture all fragments containing exons.

Whole-exome sequencing was performed on the SOLiD 5500xl system with 150 bp paired-end read. Primary analysis was performed on SOLiD instrument control software (SOLiD ICS) followed by the secondary analysis, mapping and variants calling, on LifeScope Genomic Analysis server. The human reference genome assembly hg19 (GRCh37) was used as a reference sequence in this analysis process.

**Figure 2:**
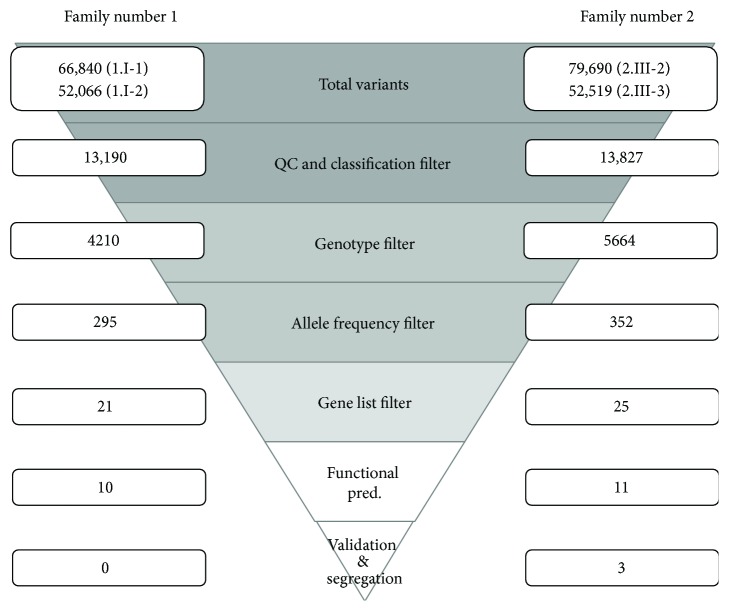
Filtering procedure of variants obtained by whole-exome sequencing 2nd data analysis. The number indicates an amount of variants passed for each step.

Tertiary analysis, which includes quality filtering, annotation, data aggregation, and multisample processing, was performed with a variant call format (VCF) file from the secondary analysis. By using Golden Helix SVS software, all variants which have genotype quality or coverage depths lower than 20 and 10, respectively, were filtered out. Noncoding and synonymous variants were removed after annotation with the UCSC KnownGenes database. High-frequency variants commonly found in the general population were filtered out by annotation with 1000 Genomes Project Phase 3 [19] and an in-house exome database which consists of 172 Thai individuals. Variants that have minor allele frequency (MAF) greater than 2% were excluded in this step. Functional prediction databases were used to assess an effect of variants on deleterious encoded protein function. The database for this process was dbNSFP NS Functional Predictions v2.3 that compiled prediction scores from several prediction algorithms including SIFT, Polyphen2, MutationTaster, LRT, MutationAssessor, GERP++, phastCons, PhyloP, FATHMM, PROVEAN, MetaSVM, MetaLR, CADD, VEST3, and SiPhy and other related information [20]. This step removed variants that were predicted not to affect protein function by any algorithms. Filtering by gene list was performed to emphasize variants on genes that are most possibly related to ASD. The gene list consisted of reported ASD-associated genes from public databases, AutDB and AutKB [21, 22]. Furthermore, brain function and signaling-associated gene panels from Enlis Genome Research software (Enlis, Berkeley, CA) were included. Finally, genotype filtering according to inheritance mode was executed. Genotypes shared between 2 siblings for each family (both homozygous for the recessive allele, both heterozygous for de novo dominant allele) were identified as candidate variants. Two or more heterozygous variants in the same gene indicate a compound heterozygous inheritance model. A summary of the steps to filter variants is shown in Figure 2. Additionally, the Human Gene Mutation Database (HGMD) was used to identify known pathogenic variants [23].

### 2.3. Variant Validations and Segregation Analysis

Candidate variants from whole-exome sequencing were validated by Sanger sequencing. Along with this validation process, segregation analyses were performed on the family members. Primer3 v0.4.0, a web-based tool, was used to designed primers for amplification and sequencing [24]. Sequencing reactions were performed using Applied Biosystems 3130 DNA Analyzer (Life Technologies, Carlsbad, CA, USA).

## 3. Results

All samples from the autistic patients were successfully sequenced and variants detected with an average depth over 60× coverage. For family number 1, 66,840 and 52,066 variants were detected in samples 1.II-1 and 1.II-2 while 79,609 and 52,519 variants were detected in samples 2.III-2 and 2.III-3 for family number 2, respectively. VCF files for samples in the same family were imported together into Golden Helix SVS software for posterior annotation and filtering. A total of 13,190 variants for family number and 13,827 variants for family number 2 remained after removal of low-quality, noncoding, and synonymous variants. Afterwards, a filtering pipeline for minor allele frequencies, inheritance models, and a candidate gene filter was executed to reduce the number of variants to 21 and 25 variants for family numbers 1 and 2, respectively. Next, variant annotation and prioritization with the deleterious function prediction database resulted in 10 variants for family number 1 and 11 variants for family number 2. These variants were indicated as candidate variants. Finally, all candidate variants underwent validation and segregation analysis by Sanger sequencing. A total of 124 reactions of amplification and sequencing were achieved. In this process, variants that had discordant results between whole-exome sequencing and Sanger sequencing or had invalid segregation result with other family members were excluded. The candidate variants which passed in the validation process are shown in Table 1. All candidate variants of family number 1 failed in this step whereas three variants of family number 2 passed and were identified as final candidate variants for ASD in this family. The list of final candidate variants is shown in Table 2.

**Figure 3:**
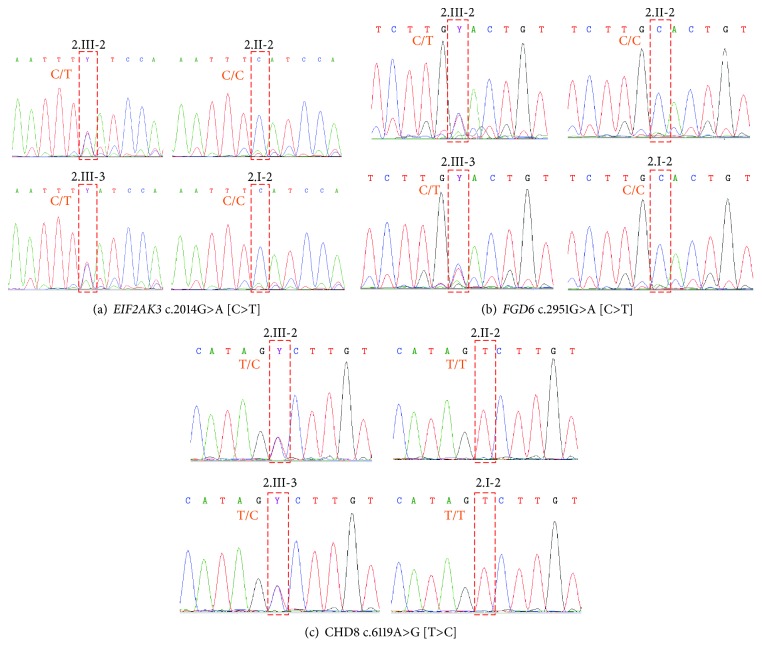
Chromatograms of 2 heterozygous missense variants in *EIF2AK3* (a), *FGD6* (b), and *CHD8* (c) gene. Letters in indicate complementary (FWD) alleles.

All three final candidate variants were nonsynonymous missense variants with genotype heterozygous in both autism-affected siblings (2.III-2 and 2.III-3) while showing homozygous wild-type in other unaffected family members. These variants consisted of c.2014G>A (NM_004836) in the *EIF2AK3* gene, c.2951G>A (NM_018351) in the *FGD6* gene, and c.6119A>G (NM_001170629) in the *CHD8* gene. Chromatograms of all variants in all available subjects are shown in Figure 3.

In addition, associations between the candidate variants and ASD were studied by using each ASD-affected individual from the family number 1 and the family number 2 plus 14 ASD-affected individuals from another project as the case group and 224 ASD-unaffected Thai individuals as the control group. Fisher's exact test was used for statistical testing in this study. The association study results revealed that only one of the three final candidate variants, c.2951G>A in the *FGD6* gene, had a statistically significant association with ASD (*P* = 0.0041). The details and results of the association study are shown in Supplementary 2.

## 4. Discussion

This study was designed to perform exome sequencing to identify possible ASD causal variants in two Thai families and successfully identified three variants for one family, family number 2. For family number 1, all 10 variants did not pass validation and segregation. This indicates that possible causal variants may be lost in the prior process. Exome capturing using beads is one factor that highly impacts loss of target fragments [25]. Moreover, the possible causal variant may be passed over if it is located within a noncoding region or in genes that are not in the gene list used in the filtering process. Replication or whole-genome sequencing will be needed for this family.

Among 3 ASD candidate genes for the family number 2, *CHD8* encodes chromodomain helicase DNA binding protein 8 which functions as a transcription repressor by transforming chromatin structure. The binding of *CHD8* product and beta-catenin motivates negative regulation of the Wnt signaling pathway, which functions essentially in vertebrate early development and morphogenesis. This gene is one of the genes that have frequently been reported to be associated with ASD. This study found one possible de novo variant: c.6119A>G results in replacement of asparagine by glycine (p.Asp2040Gly) on this gene. Rare de novo mutations in this gene have been reported in individuals with ASD [26]. A study of balanced chromosomal abnormality in autism revealed disruption of genes including *CHD8* [27]. The association between the mutation in *CHD8* and ASD has been reported previously [28, 29]. Merner et al. reported 14 variants, including a de novo frameshift mutation, p.Asn2092Lysfs∗2, that results in premature stop codon leading to the loss of 212 amino acids of the protein in *CHD8* by all exon genotyping technique of 142 autistic individuals with macrocephaly, psychopathology, infantile hypotonia, lack of major ID, and speech delay [30]. Wilkinson et al. have reported de novo loss-of-function mutations in 12 individuals with ASD by whole-exome sequencing [31]. Moreover, *CHD8* knockdown by siRNA-mediated techniques revealed that insufficiency of this gene leads to changed expression of 1715 genes involved in the neuronal development pathway and previously reported to be candidate genes for ASD [31].

*EIF2AK3* encodes eukaryotic translation initiation factor 2-alpha kinase 3, also known as protein kinase R- (PKR-) like endoplasmic reticulum kinase or PRKR-like endoplasmic reticulum kinase [32]. This study found a variant, c.2014G>A, which results in replacement of glutamine by lysine (p.Glu672Lys) on this gene. Patients who have homozygous mutations in this gene develop Wolcott-Rallison syndrome, a disorder with infant-onset diabetes mellitus, hepatic and renal dysfunction, developmental delay or mental retardation, osteopenia, and multiple epiphyseal dysplasia [33]. *EIF2AK3* mutant mice revealed multiple phenotypes consistent with cognitive and information processing deficits, which are one feature of ASD [34].

The last one, *FGD6*, encodes FYVE, RhoGEF, and PH domain-containing 6, a protein-coding factor. This study found a variant, c.2951G>A, which results in replacement of cytosine by tyrosine (p.Cys984Tyr) on this gene. *FGD6* is an important paralog of the *FGD1* gene which is associated with syndromic autism, where some patients are affected with a particular syndrome that presents as autism. Specifically, patients with Aarskog-Scott syndrome (AAS) carry rare mutations in the *FGD1* gene [35, 36].

From an additional association study of the 3 final candidate variants (Supplementary 2), we found that only the variant in the *FGD6* gene, c.2951G>A, had a statistically significant association with ASD (*P* = 0.0041) and was found only in the cases but not in the controls. This result suggests that the *FGD6* variant might be an autism-associated variant. However, this association study was performed with only a small sample, and further studies with a larger number of cases and controls are needed to verify this finding.

Additionally, since one autism-affected child, 2.III-2, of family number 2 had heart block along with ASD, a cause of this heart defect was explored in our previous publication. The previous result revealed that autism and heart block in this family are coincidental, not likely to be Timothy syndrome [18].

Although whole-exome sequencing is an effective tool for the study of genetic variation, its limitation is that it sequences only coding regions of genome. Noncoding variants and structural variants were discounted in this study. Furthermore, functional prediction of the novel variants in this study was found by bioinformatics processes. Further study is required to determine biological function.

## 5. Conclusion

In conclusion, this study revealed the utility of whole-exome sequencing and a bioinformatics analysis process for identifying possible causative variants of ASD. An additional association study of the 3 final candidate variants (Supplementary 2) found that only the variant in the *FGD6* gene, c.2951G>A, had a statistically significant association with ASD. This suggests that the *FGD6* variant might be an ASD-associated variant. However, we performed the association study in only a small number of cases and controls; therefore, further studies in larger samples are needed to verify this finding.

## Figures and Tables

**Table 1:** Candidate variants from whole-exome sequencing which passed in the validation process. The variants which passed in the segregation process are presented with bold letters.

Position	Gene	Classification	Transcript	HGVS Coding	rsID 138	Family
Chr2: 73675228	*ALMS1*	Ins	NM_015120	c.1570_1571insCTC	rs34628045	1, 2
**Chr2: 88876094**	***EIF2AK3***	**Nonsyn SNV**	**NM_004836**	**c.2014G>A**	**rs35226268**	**2**
Chr4: 84240515	*HPSE*	Nonsyn SNV	NM_001199830	c.481A>C	rs75865093	1
Chr5: 140183237	*PCDHA3*	Nonsyn SNV	NM_031497	c.2455A>G	rs17844265	2
Chr5: 156721864	*CYFIP2*	Frameshift Ins	NM_001037333	c.279_280insC	rs5872508	1, 2
Chr6: 132270417	*CTGF*	Frameshift Del	NM_001901	c.1037delG	rs373467469	2
Chr7: 82581489	*PCLO*	Ins	NM_033026	c.8780_8781insTGA	rs10630259	1
Chr9: 117853022	*TNC*	Frameshift Del	NM_002160	c.276delG	rs944510	1
**Chr12: 95531341**	***FGD6***	**Nonsyn SNV**	**NM_018351**	**c.2951G>A**		**2**
**Chr14: 21861835**	***CHD8***	**Nonsyn SNV**	**NM_001170629**	**c.6119A>G**	**rs148494847**	**2**
Chr17: 26101336	*NOS2*	Nonsyn SNV	NM_000625	c.1423G>A		2
Chr21: 40883672	*SH3BGR*	Ins	NM_001001713	c.356_357insAGA	rs3831201	1

**Table 2:** List of final candidate variants.

Genomic coordinates	Genotype^∗^				AA change	Gene	MAF		Functional prediction		
	2.III-2	2.III-3	2.II-2	2.I-2			1 kG (ASN)	Thai	SIFT	Polyphen2 HumVar	MutationTaster
Chr2: 88876094	C/T	C/T	C/C	C/C	p.Glu672Lys	*EIF2AK3*	0.02	0.0067	Tolerated	Benign	Disease causing
Chr12: 95531341	C/T	C/T	C/C	C/C	p.Cys984Tyr	*FGD6*	0	0	Tolerated	Probably damaging	Disease causing
Chr14: 21861835	C/T	C/T	T/T	T/T	p.Asp2040Gly	*CHD8*	0.02	0.0067	Tolerated	Benign	Disease causing

^∗^Genotypes in this table are FWD genotype while genotypes in HGVS are REV genotype.

## Supplementary Materials

Supplementary 1 Clinical information of the study families.

Supplementary 2 Details and results of the preliminary association study.
