# Supplementary material for: Whole-Exome Sequencing Identifies One De Novo Variant in the FGD6 Gene in a Thai Family with Autism Spectrum Disorder
Source: Int J Genomics. 2018 May 17;2018:8231547. doi: 10.1155/2018/8231547 (PMC5985066; doi:10.1155/2018/8231547)
Supplement: Supplementary 1 — Clinical information of the study families. [file 8231547.f1.docx]

**Supplementary 1**

**Clinical information**

The DSM-5 was released in May 2013, but the two case reports were recruited before 2013, therefore DSM-5 was not available. The clinical information in the current whole exome sequencing analysis was based on previously reported [1, 2]. The ADOS and ADI-R (Thai version) were not available at the time we did the current study, and the English versions were not commonly used in clinical practice in Thailand. We developed and used a new structured interview for Thai children with ASD for research based on the DSM-IV [3].

The two probands were evaluated for the common genetic causes of ASD and had negative DNA tests for Fragile X syndrome (both families) and *MECP2* mutations (family no. 2), and normal chromosome studies by karyotyping [1] and microarray (Infinium CytoSNP-850K v1.1 Beadchip, Illumina, San Diego, California, USA) [4].


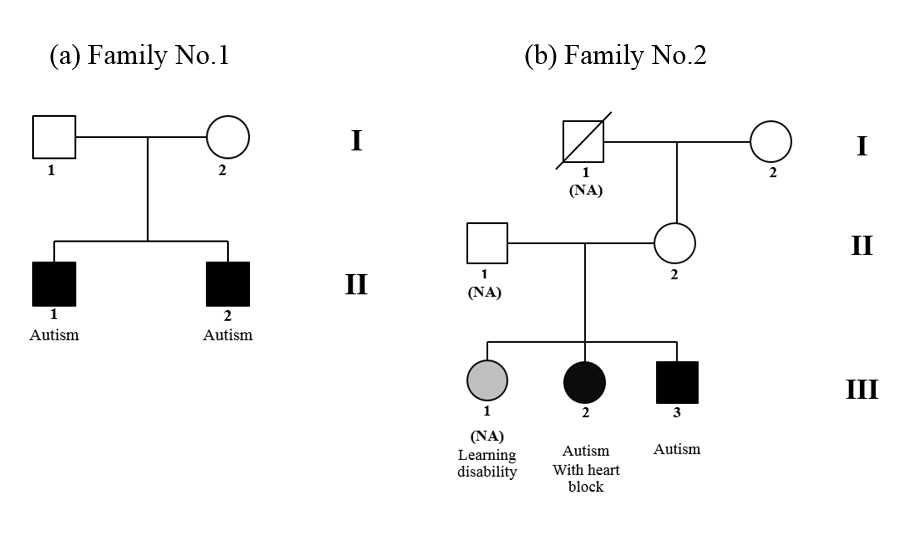


**Family 1**

The proband (1.II-2) was brought to the clinic because of delayed language development. He was born by vaginal delivery without complications with birth weight of 3,689 grams. A physical examination at 4 years of age found neither dysmorphic face nor abnormal skin pigmentation. His weight and height were 21.3 kg (90-97 centile) and 108.5 cm (90-97 centile), respectively. His head circumference was 51.5 cm (90 centile). He had poor eye contact and was hyperactive. He could speak only 5 words. Non-verbal IQ (Stanford-Binet Intelligence Scale: fifth edition, SB:V) was 43 (moderate impairment). Behavior and social evaluation using the Vineland Adaptive Behavior Scales (Interview edition survey form) showed that he had moderately low to low adaptive levels in all domains including communication, daily living skills, socialization and motor skills. He was diagnosed as having autism using a structured interview [1]. His older brother was also diagnosed as having autism. However, his brother had mild clinical features with a higher non-verbal IQ score (SB:V) of 92 and showing only mild deficit in the Vineland Adaptive Behavior Scales.

**Family 2**

The proband (2.III-2) had a third-degree atrioventricular block (AV block) and had undergone cardiac surgery for epicardial pacemaker implantation at the age of 18 months [3]. She had been observed to have developmental delay since she was 1 year of age. Due to her heart condition and family problems, her developmental delay was not recognized during this time and thus medical help was not sought. According to the mother, she could speak one word at approximately 2 years of age and after that, she could speak a few short sentences and some jargon. She was evaluated by a child developmental pediatrician (DT- author) at 6 years of age when she had to attend a special school. Her body weight and height were 32 kg (> 97 centile) and 125 cm (> 97 centile), respectively. Her head circumference was 54 cm (75-90 centile). Her general appearance looked normal but she was hyperactive. She was diagnosed as having autism using the DSM-IV without a structured interview for initial evaluation. Then she was re-evaluated by a structured interview [1] to confirm a diagnosis of autism. The other two siblings had no heart defects but the older sister (3.III-1) had a learning disability and the younger brother (2.III-3) had autism according to DSM-IV criteria. Further analysis was prevented when the proband was too uncooperative to do an IQ test or Vineland Adaptive Behavioral Scales.

**References**

[1] C. Charalsawadi, W. Maisrikhaw, V. Praphanphoj et al., "A case with a ring chromosome 13 in a cohort of 203 children with non-syndromic autism and review of the cytogenetic literature," *Cytogenetic and Genome Research*, vol. 144, no. 1, pp. 1-8, 2014.

[2] C. Thongnak, P. Limprasert, D. Tangviriyapaiboon et al., "Exome Sequencing Identifies Compound Heterozygous Mutations in SCN5A Associated with Congenital Complete Heart Block in the Thai Population," *Disease Markers*, vol. 2016, pp. 3684965, 2016.

[3] T. Hansakunachai, R. Roongpraiwan, T. Sombuntham, P. Limprasert and N. Ruangdaraganon, "A new structured interview for children with autism spectrum disorder based on the DSM-IV," *Journal of the Medical Association of Thailand*, vol. 97, (Suppl 8), pp. S7-14, 2014.

[4] A. Hnoonual, W. Thammachote, T. Tim-Aroon et al., "Chromosomal microarray analysis in a cohort of underrepresented population identifies *SERINC2* as a novel candidate gene for autism spectrum disorder," *Scientific Reports*, vol. 7, no. 1, pp. 12096, 2017.
