# Supplementary material for: Whole-Exome Sequencing Identifies One De Novo Variant in the FGD6 Gene in a Thai Family with Autism Spectrum Disorder
Source: Int J Genomics. 2018 May 17;2018:8231547. doi: 10.1155/2018/8231547 (PMC5985066; doi:10.1155/2018/8231547)
Supplement: Supplementary 2 — Details and results of the preliminary association study. [file 8231547.f2.docx]

**Supplementary 2**

**Preliminary association study**

We performed a preliminary association study in 14 additional ASD cases (4 unpublished and 10 published cases) [1]. Therefore, in the end, we had 16 ASD cases (2 from this study and the additional 14 cases) and 224 non-ASD controls for the association study. The results are shown in Supplementary Table 1.

**Supplementary Table 1** Association study results of the 12 valid candidate variants. The 3 variants of the *EIF2AK3, FGD6,* and *CHD8* genes are variants in the final candidate list. Bold type indicates statistical significance at *P*-value (**).

| **Position** | **Gene** | **Ref** | **Alt** | Case  (16) | Control  (224) | **Fisher's exact *P-*value** | **Odd ratio** | **95% CI of Odd** |
| --- | --- | --- | --- | --- | --- | --- | --- | --- |
| Chr2: 73675228 | *ALMS1* | - | CTC (ins) | 4 | 86 | 0.4236 | 1.7117 | (0.1671 - 1.7117) |
| Chr4: 84240515 | *HPSE* | A | C | 1 | 2 | 0.1877 | 86.3379 | (0.6343 - 86.3379) |
| Chr5: 140183237 | *PCDHA3* | A | G | 1 | 0 | 0.0667 | na | na |
| **Chr5: 156721864** | ***CYFIP2*** | **-** | **C (ins)** | **4** | **167** | **0.0001**** | **0.3669** | **(0.0353 - 0.3669)** |
| Chr6: 132270417 | *CTGF* | G | - (del) | 1 | 1 | 0.1291 | 249.5987 | (0.8855 - 249.5987) |
| Chr7: 82581489 | *PCLO* | - | TGA (ins) | 2 | 63 | 0.2475 | 0.3650 | (0.0807 - 1.6526) |
| Chr9: 117853022 | *TNC* | G | - (del) | 2 | 22 | 0.6660 | 1.3117 | (0.2796 - 6.1528) |
| Chr17: 26101336 | *NOS2* | G | A | 1 | 0 | 0.0667 | na | na |
| **Chr21: 40883672** | ***SH3BGR*** | **-** | **AGA (ins)** | **3** | **133** | **0.0028**** | **0.5698** | **(0.0438 - 0.5698)** |
| Chr2: 88876094 | *EIF2AK3* | G | A | 2 | 7 | 0.1140 | 4.4286 | (0.8405 - 23.3340) |
| **Chr12: 95531341** | ***FGD6*** | **G** | **A** | **2** | **0** | **0.0041**** | **na** | **na** |
| Chr14: 21861835 | *CHD8* | A | G | 2 | 9 | 0.1611 | 3.4127 | (0.6721 - 17.3273) |

Ref = Reference allele, Alt = Alterative allele, na = not available, ins = insertion, del = deletion

Among the 12 variants from the candidate variants list, 3 variants had a significant *P*-value (*P* < 0.05). Among these 3 variants, chr12:95531341 (A allele, c.2951G>A) in the *FGD6* gene is a statistically significant variant since it was found only in the cases and not in the controls (*P* = 0.0041). The other 2 variants, chr5:156721864 and chr21:40883672 in the *CYFIP2* and *SH3BGR* genes, respectively, also had statistically significant *P*-values but associations with ASD were not considered because the alterative alleles were found in large numbers in the controls. Moreover, these two variants were found in unaffected individuals in the family study. It should be noted that we performed the association study from a small number of cases and controls, therefore further studies with larger samples need to be done to verify this finding.

**Reference**

[1] A. Hnoonual, T. Sripo and P. Limprasert, "Whole-exome sequencing identifies a novel heterozygous missense variant of the *EN2* gene in two unrelated patients with autism spectrum disorder," *Psychiatric Genetics*, vol. 26, no. 6, pp. 297-301, 2016.
